# A digital intervention to protect cognitive health in older adults with cognitive impairment: a three-arm, open-label, randomised controlled trial

**DOI:** 10.1016/j.lanprc.2026.100192

**Published:** 2026-08

**Authors:** Paul Little, Rosie Essery, Jane Somerville, Taeko Becque, Victoria Hayter, Beth Stuart, Katherine Bradbury, Gareth Griffiths, Simon Griffin, Tony Kendrick, Nanette Mutrie, Sian Robinson, Louise Robinson, Guiqing Yao, Shihua Zhu, Sebastien Pollet, James Denison-Day, Helen Brooker, Anne Corbett, Paul Lee, Shanaya Rathod, Bernard Gudgin, Rosemary Phillips, Tom Stokes, John Niven, Angela McCullagh, Sara McCloskey, Martin Rossor, John Gallacher, John O’Brien, Clive Ballard, Lucy Yardley

**Affiliations:** a Primary Care Research Centre, Faculty of Medicine, University of Southampton, Southampton, UK; b School of Psychology, University of Southampton, Southampton, UK; c Southampton Clinical Trials Unit, University of Southampton and University Hospital Southampton NHS Foundation Trust, Southampton, UK; d Department of Psychiatry, School of Clinical Medicine, University of Cambridge, Cambridge, UK; e Department of Public Health and Primary Care, University of Cambridge, Cambridge, UK; f Physical Activity for Health Research Centre, University of Edinburgh, Edinburgh, UK; g Wolfson Institute of Population Health, Queen Mary University of London, London, UK; h Population Health Sciences, Newcastle University, Newcastle, UK; i Cardiovascular Sciences, University of Leicester, Leicester, UK; j Exeter Biomedical Research Centre, University of Exeter, Exeter, UK; k Hampshire and Isle of Wight Healthcare NHS Foundation Trust, Southampton, UK; l Patient and Public Involvement Contributor, University of Southampton, Southampton, UK; m Queen Square Institute of Neurology, University College London, London, UK; n Department of Psychiatry, University of Oxford, Oxford, UK; o School of Psychological Science, University of Bristol, Bristol, UK; p Ecog Pro, Bristol, UK

## Abstract

**Background:**

Cognitive impairment affects health and social care. Intensive multicomponent interventions work but are impractical for primary care. The study aimed to trial brief approaches for cognitive health.

**Methods:**

This was an open-label trial among adults aged 60–85 years with internet access and low cognitive scores (SD ≥1 below normal on the Baddeley verbal reasoning test, the cognitive criteria for age-associated-cognitive decline [AACD]). We used computer-generated random numbers to allocate participants to one of three groups: (1) Active Brains website (supporting physical activity, a Mediterranean diet, and cognitive exercises); (2) Active Brains plus brief support (three email or telephone sessions); or (3) control (evidence-based advice for cognitive health). The primary outcome was Baddeley verbal reasoning score at 12 months analysed by intention-to-treat with multiple imputation for missing data; follow-up for a co-primary outcome of dementia at 5 years is ongoing. The key secondary outcome was to describe the clinically important cutpoints of the Baddeley test. The trial is registered with International Standard Randomised Controlled Trial (ISRCTN17349359), is closed to new participants, and follow-up is ongoing.

**Findings:**

Between Oct 3, 2020, and Oct 30, 2023, 11 563 participants from 670 practices were randomly assigned to either the active brains group (n=3169), active brains plus support (n=3087), or control (n=3927; intention-to-treat population). Participants were predominantly White (11 087 [95·8%]), 6697 (57·9%) were female, and 8142 (70·4%) were retired. Recruitment and 12-month follow-up (median 366 days in all groups) are complete. Baseline mean Baddeley verbal reasoning score was 15·1 (SD 6·4) in the control group, 15·3 (6·2) in the Active Brains group, and 15·1 (6·4) in the Active Brains plus support group. 12-month data were available for the primary outcome for 3113 (79·3%) of 3927 participants in the control group, 2564 (67·3%) of 3810 in the Active Brains group, and 2566 (67·1%) of 3826 in the Active Brains plus support group. At 12 months, mean Baddeley verbal reasoning score was 18·3 (SE 0·14) in the control group, 18·7 (SE 0·16) in the Active Brains group (adjusted mean difference *vs* control +0·50, 95% CI 0·13–0·87), and 19·2 (SE 0·16) in the Active Brains plus support group (+0·97, 0·59–1·34). This resulted in a substantial minority in all groups no longer meeting the cognitive criteria for AACD (883 [28·4%] of 3113 in the control group; 842 [32·8%] of 2566 in the Active Brains group; 811 [31·6%] of 2546 in the Active Brains plus support group), and fewer in the Active Brains plus support group (but not the Active Brains group) met cognitive criteria for mild or severe cognitive impairment. Serious adverse events and deaths, all unrelated to treatment, were similar in all groups. Serious adverse events occurred in 33 (0·87%) of 3810 in the Active Brains group, 32 (0·84%) of 3826 in the Active Brains plus support group, and 28 (0·71%) of 3927 in the control group. Deaths occurred in 20 (0·5%) in the Active Brains group, 17 (0·4%) in the Active Brains plus support group, and 19 (0·5%) in the control group.

**Interpretation:**

Very brief intervention (eg, cognitive testing, documenting brain-stimulating activities, and evidence-based advice on brain health) in primary care is likely to improve cognitive health among individuals with low cognitive functioning. This study further supports the additional use of scalable online interventions for cognitive impairment in that require minimal support. Research is needed to assess the longer term effects of these interventions.

**Funding:**

National Institute for Health and Care Research Programme Grants for Applied Research.

## Introduction

Dementia causes disability and dependency among older adults, and costed an estimated US$1·3 trillion globally in 2019, according to WHO. In response, WHO extended its 2025 global action plan for dementia to 2031.^1^ Among adults older than 60 years, up to 18% have mild cognitive impairment (MCI),^2^ with a similar number having age-associated cognitive decline (AACD), and with an estimated 10% of those with MCI and AACD progressing to dementia annually.^3^ Scalable, evidence-based approaches to modifying cognitive decline are crucially important for public health.

**Research in context**

**Evidence before this study**

The UN recognises cognitive impairment as a non-communicable disease. Intensive multidomain interventions improve cognition but are not pragmatic for primary care. A meta-analysis of briefer web-based interventions, which searched for cognitive interventions using PubMed, Embase, and PsycINFO, from database inception until June 5, 2018, using search terms ("Cognition"/"Cognitive Aging"[Mesh]/"Memory"[Mesh]) OR brain health[tiab]; and lifestyle ("life style"[mesh] OR "health behavior"[mesh] OR "Stress, Psychological"[Mesh] OR "exercise"[mesh]) OR multimodal[tiab]/multidomain[tiab]), found encouraging results, but had a short-term follow-up or significant additional input. The Maintain Your Brain intervention over 3 years found similar results to FINGER but did not recruit from primary care.

**Added value of this study**

The added value of this study is in showing the use of a range of very brief interventions for the key population (aged 60–85 years with low cognitive scores), recruited in primary care, and not needing face-to-face appointments: firstly, the effect of cognitive testing and evidence-based advice alone in reducing individuals meeting the criteria for age-associated cognitive decline; and secondly, the effect of online interventions (eg, addressing physical activity, Mediterranean diet, and brain training) with brief support (an 8% relative reduction in those meeting cognitive criteria for mild cognitive impairment [risk ratio 0·92; 95% CI 0·87–0·97]), and for individuals with the cognitive criteria for mild cognitive impairment at baseline, an 18% reduction in cognitive scores consistent with severe cognitive impairment [risk ratio 0·82; 0·70–0·96]) at 12 months.

**Implications of all the available evidence**

This study supports the use of brief, scalable online interventions that require minimal support in primary care to improve cognitive health among individuals with low cognitive functioning. Research is needed to assess the longer-term impact of these interventions, and the impact in individuals with normal cognitive functioning.

40% of risk factors for dementia are potentially modifiable for preventing or slowing disease.^4^ The FINGER trial (n=1260), targeting diet, physical activity, cognitive training, and vascular risk monitoring, resulted in a modest effect on cognitive decline, which is being replicated across diverse populations,^5^ as shown by the most recent results of the US POINTER trial.^6^ A meta-analysis of multidomain interventions found benefit in ten of 16 studies, but most involved intensive interventions and with short-term outcomes.^7^ Addressing the size of the target population with intensive face-to-face programmes is prohibitively resource-intensive and not pragmatic in primary care, nor is it feasible for low-income and middle-income countries, and although modest benefits in cognition have been reported, the clinical relevance, as evidenced by progression to MCI or dementia, has not been established.

To enable wider implementation and to achieve benefits at a population level, more scalable interventions are required for primary care. A meta-analysis of digital interventions showed small-to-medium effects for briefer more focused approaches.^8^ Since only three of the 14 studies in the meta-analysis had control groups, robust pragmatic randomised controlled trial evidence is needed. Furthermore, none of the three controlled trials reported outcomes beyond 3 months.^8^ Promising results were found over 6 months with remotely delivered cognitive training,^9^ and the 3-year follow-up of the Maintain Your Brain online intervention (which provided both user-generated access to supervision and supervisor feedback for poor performance) found similar effects to the FINGER trial, and suggests that scalable approaches could possibly work over a longer time period.^10^

We report the 12-month follow-up of a fully powered trial of a robustly developed multidomain intervention (Active Brains website).^11^ The objective was, among those with lower cognitive performance, to estimate the benefit on cognitive performance, including on mild and severe cognitive impairment, of a multifaceted but brief, remotely delivered, potentially scalable intervention targeting physical activity, healthy eating behaviours, and online cognitive training.

## Methods

### Study design and participants

This study is an open-label, parallel group, randomised controlled trial, registered as an International Standard Randomised Controlled Trial (ISRCTN17349359) on April 28, 2020. There were two populations of interest: individuals with low cognitive functioning (a low cognitive score meeting the cognitive criteria for AACD) and with normal cognitive functioning (a normal cognitive score, thus not meeting the cognitive criteria for AACD).

Allocation to each category was determined by the participants’ baseline scores on a computerised Baddeley Verbal Reasoning task.^12^ Reasoning is a key domain as it is sensitive to cognitive training, and a key mediator in maintaining instrumental activities of daily living (IADL).^13^ The Baddeley Verbal Reasoning task was also a suitable screening tool, drawing on the extensive PROTECT database of scores to define clinically important cutpoints, which are normative scores meeting the cognitive criteria for AACD, and scores consistent with the cognitive criteria for MCI and the cognitive criteria for severe cognitive impairment (SCI).

We report the 12-month follow-up of the trial among participants with lower cognitive scores, and a 5-year follow-up is planned. The trial among participants with normal cognitive scores will be reported separately. Once we reached the target sample size for those with normal cognitive scores, further individuals with normal scores were allowed access to the Active Brains website (cohort group), which will not be reported here.

Primary care practices in England, Wales, and Scotland were initially approached by their regional Clinical Research Network. Practices that agreed to participate (and they could withdraw at any time) undertook medical records database searches, screening, and mailout, recording age, sex, and postcode of invitees. Mailouts provided access to the Active Brains study website, where potential participants could sign up, consent, and complete screening (including the Baddeley test). Eligible participants could then complete baseline measures followed by randomisation.

Adults aged 60–85 years were included in the study. This age restriction was chosen because people in this age group are typically more concerned about the development of dementia in themselves and their partners. We aimed to include most individuals with cognitive decline (older than 60 years), and although internet access is growing rapidly, few participants older than 85 years have home access, hence these individuals were excluded.

Those with an SD of ≥1 below the norm for the Baddeley reasoning test,^12^ who met the cognitive criteria for AACD, were included. Normative scores were determined from the PROTECT cohort database^14^ (n=10 714) of older adults’ data on a battery of cognitive assessment tasks. Within this group, the cognitive criteria for MCI was defined as an SD of ≥1·5 below the normative scores and the cognitive criteria for SCI was defined as an SD of ≥2 below the normal scores for the Baddeley reasoning test.^12^

The exclusion criteria were limited scope for intervention benefit (eg, existing diagnosis of dementia; or high levels of leisure time physical activity with a score of 30 or more on the Godin Leisure Time Exercise Questionnaire);^15^ limited ability, or too unwell, to consent to or access the intervention (eg, terminal care; poorly controlled major mental health problems [eg, major depressive illness]; or no or limited internet access); or interference with or contamination of the intervention group (eg, diagnosis of post-COVID-19 condition; self-reported participation in another intervention to support cognitive health; or another member of household already participating).

This study was approved by Yorkshire & The Humber–Bradford Leeds Research Ethics Committee (20/YH/0018) and the Heath Research Authority on Feb 24, 2020. All participants provided written informed consent via the Active Brains portal. Although we changed the order of the verbal reasoning tasks halfway through the trial to ensure we collected the primary outcome as robustly as possible, the order was never specified in the protocol. Other protocol changes are listed in the appendix (p 17). There were two protocol deviations, both of which were appropriately investigated and reported according to the Sponsor’s Corrective Actions and Preventative Actions reporting process. Both were assessed as minor incidents. Five public contributors provided key input at all stages of the study: in developing the protocol, study processes, study materials, and the intervention, and subsequently in the ongoing management of the study, interpretation of data, and revising the paper for publication (appendix p 18).

### Randomisation and masking

Following online consent participants were randomly assigned (simple randomisation, with no strata) by bespoke online software that used computer-generated random numbers (developed by Global Initiative) in a 1:1:1 ratio to one of three groups: Active Brains digital intervention, Active Brains digital intervention plus support, or control.

Before randomisation, participants were masked to the intended group, but this was not possible after randomisation. The research team making any follow-up phone calls were masked to group, as were the statisticians when performing the analysis.

### Procedures

All groups had cognitive testing and questionnaires about brain-stimulating activities at baseline and 12-month follow-up. Other than this all groups were able to access medical care as they would have normally, and medical care was not constrained.

Participants assigned to the Active Brains group were given access to the Active Brains website, which provides tailored support to encourage older adults to engage in behaviours that are beneficial to their cognitive and physical health. These include supporting physical activity (eg, addressing overall levels of physical activity, reducing sedentary behaviour, and strength and balance exercises), healthy eating (Mediterranean diet), and cognitive exercises (brain-training: providing up to 12 games over 6 months from the PROTECT-UK website). Additional details of the Active Brains intervention are available in the appendix (pp 1–4). Those assigned to the Active Brains plus support group also had access to the digital intervention and also offered three brief (approximately 10 min) telephone support sessions, with more if needed. These support sessions were either with research nurses based in their participating practice or research network, or University of Southampton employees with central facilitators. Participants in the control group received a single page evidence-based advice sheet about activities to protect cognitive health, as well as usual care.

At baseline, participants self-reported demographic details (ie, age, date of birth, gender, postcode [to estimate deprivation indices], internet experience, education level, work status, ethnicity, height, and weight) and all outcome measures online. Participants were invited via email to complete online follow-up measures at 1 year. A 5-year follow-up is planned, but only the 1-year data are reported here (the protocol and full details of data collection have been published online).^16^ Participants who had not completed the online cognitive assessment tasks (comprising the Baddeley Verbal Reasoning task [primary outcome], the digit span task, the paired associates learning task, and the self-ordered search task), the IADL, and the EQ5D-5L measures after 3 weeks (including two reminder emails) were contacted by paper mailout and, where necessary, by phone to prompt completion of the measures. The cognitive assessment tasks could only be completed online. If the primary outcome remained incomplete after 8 weeks with no participant contact, emails and phone calls were made to the participant’s nominated contact person (if provided). These requested that the contact person prompted or supported the participant to complete the measures or, if that was not possible, for the contact person to complete the short form of the Informant Questionnaire on Cognitive Decline in the Elderly (IQCODE SF) and proxy versions of the IADL and EQ-5D-5L.

Data on adverse events, serious adverse events, and deaths were reported by both patients and participating practices, and their relation to study treatment determined by the chief investigator.

As part of an implementation assessment, with informed consent, we analysed all website usage, which was unobtrusively and automatically collected by the LifeGuide system (the software used to build and host the Active Brains digital intervention),^17^ including time spent on each page and user-entered data (eg, goal setting and goal-related progress in all behaviours).

### Outcomes

The primary outcome at 12 months was the Baddeley verbal reasoning score, a validated test of executive function.^12^^,^^14^ The test measures how many logical statements (up to 64) a participant can correctly answer in 3 min. In the online version used, each question presents a logical premise involving the shapes “square” and “circle” using the adjectives “bigger” or “smaller” or verb phrases “encapsulates” or “is encapsulated by”. Beneath the statement is an image containing a square and circle. The participant must decide if the arrangement or relative size of the shapes within the image accurately satisfies the statement. Participants click true or false (or yes or no). The co-primary outcome was the diagnosis of dementia (to be reported at 5 years).

The secondary outcomes are stated in the protocol and the statistical analysis plan.^16^ The key secondary outcome was to describe clinically important groups defined by cutpoints in the Baddeley test—ie, meeting the cognitive criteria for MCI (SD ≥1·5 below normal); or from having had a cognitive criteria for MCI at baseline, and meeting the cognitive criteria for SCI (SD ≥2 below the normal). Instrumental Activities of Daily Living was another key secondary outcome.

The secondary cognitive outcomes were Spatial Working Memory measured through the widely used self-ordered-search test; Numerical Working Memory measured through a version of the digit span task; and verbal short-term memory measured through the paired associates learning.

Other secondary outcomes were the self-reported IQCODE SF; patient enablement measured via the modified Patient Enablement Index; depression (assessed via the short form of the Geriatric Depression Scale); health-related quality of life measured via EQ5D; 12-Item Short Form Health Survey; and the Short Warwick Edinburgh Mental Well-Being Scale.

Process measures reported here are Problematic Experiences of Therapy Scale; Self-Efficacy for Exercise Scale; Locus of Causation in Exercise; measures of key behaviours (dietary behaviour;^18^ and physical activity measured using the International Physical Activity Questionnaire modified for the elderly [IPAQ-E]); and brain training behaviours.

A full process analysis is forthcoming, which uses a wider range of measures (including the Technology Acceptance Model, Perceived Ease of Use scale, Pedometer use or purchase, strength and balance items, the social support survey, and support for the Exercise scale).

Resource usage (eg, medication, consultations, hospitalisation, accident and emergency department attendance, or outpatient visits) will be extracted through notes review for the 5-year follow-up for a planned health economic analysis; dementia was self reported for the current (1 year) results, and adverse outcomes (serious adverse events and death) were reported by both participants and practice staff).

### Statistical analysis

For the sample size, our null hypotheses were that there would be no differences in Baddeley score between the Active Brains plus support group and control group and between the Active Brains group and control group. Assuming α=0·05 and 80% power for comparison of each intervention with control, we estimated we needed 6980 participants for the Baddeley outcome at 12 months and 10 940 for the dementia outcome at 5 years. A more detailed description of the sample size calculation is in the appendix (p 18).

The intention-to-treat (ITT) analyses were done for all outcomes, using Stata version 17.0. The ITT population included all participants randomly assigned to treatment groups. The Baddeley verbal reasoning score and other continuous outcomes were analysed by use of linear regression, adjusting for baseline values, age, sex, index of multiple deprivation score, number of comorbidities and polypharmacy (yes or no); at follow-up, mean estimates were presented with standard errors (SE). The primary ITT analysis used multiple imputation with chained equations, with a complete cases sensitivity analysis. The imputation model included all variables in the analysis model and any baseline covariates predictive of outcome or its missingness, ascertained by use of Lasso regression. 100 imputations were performed and the imputed estimates combined with Rubin’s rules. Imputation was performed separately by each randomised group and then combined. The primary outcome was imputed, but not the secondary outcomes, consistent with the Statistical Analysis Plan. Dichotomous outcomes (eg, the key secondary outcomes of poor cognitive performance; ie, individuals meeting cognitive criteria for AACD criteria now fulfilling cognitive criteria for MCI and those fulfilling the cognitive criteria for MCI now meeting cognitive criteria for SCI) were analysed by logistic regression, adjusting for key baseline variables as for the primary analysis. All tests were two-tailed, with point estimates and 95% CIs for the treatment effect presented.

For the primary outcome at 1 year, exploratory subgroup analyses were performed by repeating the primary analysis and including a treatment by covariate interaction term for the following baseline subgroups: age (older or younger than 75 years); sex (male or female); deprivation based on postcode (index of multiple deprivation score: more deprived [deciles 1–5] *vs* less deprived [deciles 6–10]); comorbidities (<2 *vs* ≥2); polypharmacy (number of medications ≥5 *vs* ≤4); healthy diet (higher [upper quartile] or lower prudent diet score [food frequency questionnaire]); physical activity (higher [upper quartile] or lower IPAQ-E score); depression (depression *vs* no depression, based on Geriatric Depression Scale at baseline); retirement (retired or working); and ethnicity (White *vs* ethnic minority).

In exploratory sensitivity analyses, we explored the effect of the intervention on participants as follows: using broader definitions of AACD and MCI (since AACD and MCI definitions vary and commonly include poor functioning on more than one domain); the sensitivity to missing at random assumptions; and the effect of changing the order of the verbal reasoning tasks (which we did halfway through the trial to ensure we collected the primary outcome as robustly as possible).

Complier Average Causal Effects estimates were obtained using instrumental variables regression estimated using two stage least squares, with randomisation as the instrument and treatment receipt as the endogenous variable. Estimation was carried out separately for each of the two intervention groups and adjusted for the same covariates as in the primary analysis. Treatment receipt was defined as completing the first session of Active Brains and adhering to some of the recommended behaviours or accessing brain training more than once, based on our experience of sufficient engagement for previous digitally supported interventions. The risk ratios for serious adverse events were determined using logistic regression.

We documented the effect of clustering at a practice level by included a random effect for practice. As a post-hoc analysis, we also recorded improvement in cognitive functioning where individuals no longer met the cognitive criteria for AACD.

### Role of the funding source

The funder of the study had no role in study design, data collection, data analysis, data interpretation, writing of the report.

## Results

From Oct 3, 2020, to Oct 30, 2023, 11 563 participants from 670 practices, who were found to have low cognitive scores on the Baddeley verbal reasoning test, were recruited and randomly assigned. The mean number of participants recruited per site was 17·3 (median 14 [range 0–119]). Data collection for the 12-month follow-up was complete and signed off on March 1, 2025. Missing data are provided (figure) for the primary outcome for participants in the control group (814 [20·7%] of 3927), with higher withdrawal rates from the Active Brains group and Active Brains plus support group (data missing for 1246 [32·7%] of 3810 and 1260 [32·9%] of 3826, respectively). There were very few differences in baseline characteristics according to missingness (table 1; appendix p 16). The primary analysis uses imputed data, which minimises the effect of any potential attrition bias. The similarity of estimates from complete cases versus imputed data suggest minimal attrition bias. As expected for this age group, participants were predominantly White (5% from ethnic minority backgrounds would be expected based on the 2021 census data for this age group) and mostly retired. There was a wide range of educational attainment, slightly more female than male participants, a minority living alone, and a minority who reported being depressed. Slightly more than half were engaged with brain-stimulating activities at least a few times a week, and there were good scores for wellbeing, social support, and self-efficacy for exercise. Participants were already physically active (based on the self-reported IPAQ questionnaire). Groups were well balanced for all variables.

**Figure:**
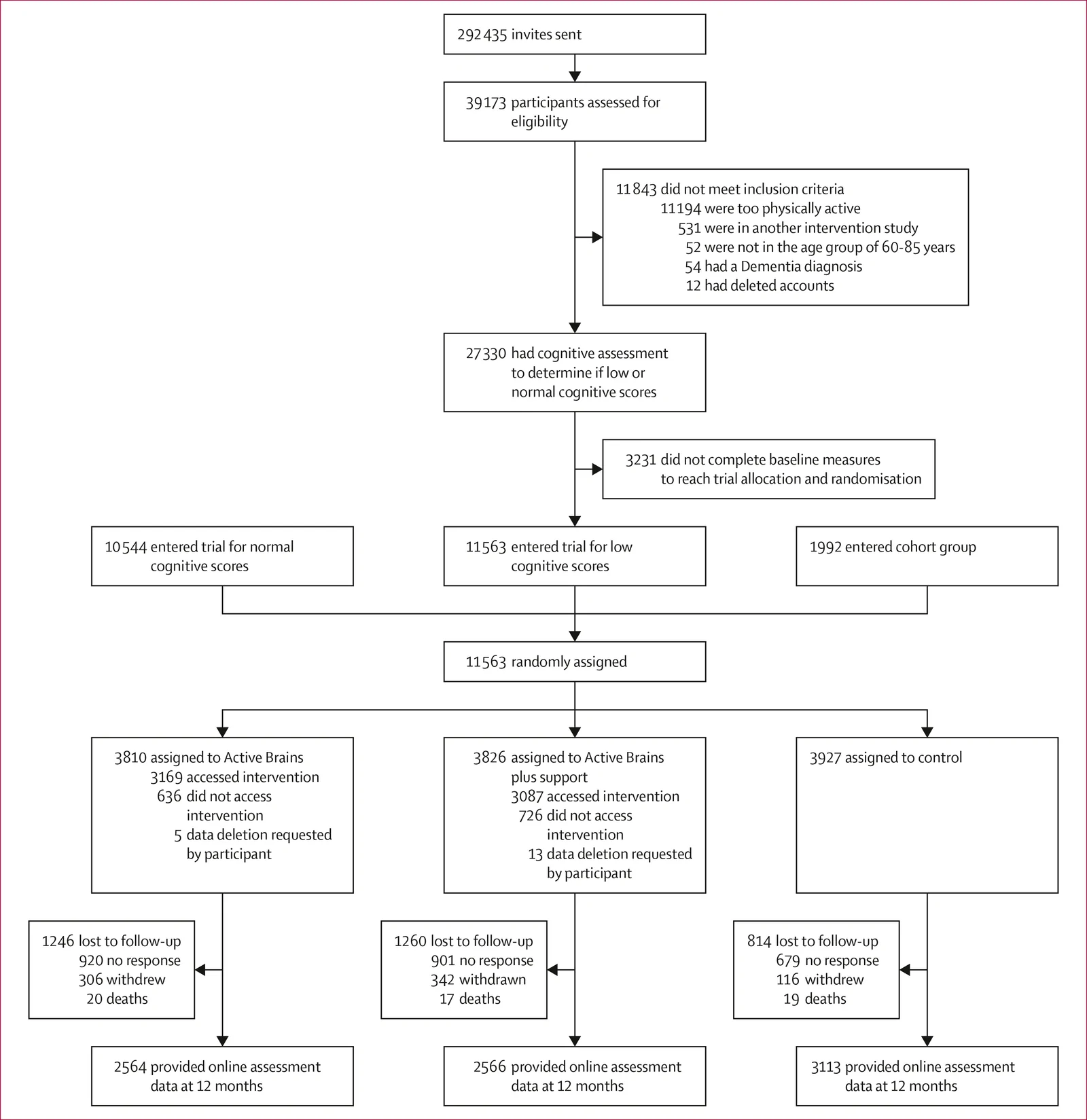
Trial profile

**Table 1:** Baseline characteristics

	Active Brains group (n=3810)	Active Brains plus support group (n=3826)	Control group (n=3927)
Age, years	70·8 (6·5)	70·8 (6·5)	70·9 (6·5)
Data missing	7 (0·2%)	13 (0·3%)	5 (0·1%)
Sex			
Male	1601 (40·8%)	1680 (44·1%)	1585 (41·7%)
Female	2319 (59·2%)	2133 (55·9%)	2220 (58·3%)
Data missing	7 (0·2%)	13 (0·3%)	5 (0·1%)
Ethnicity			
White	3757 (95·8%)	3673 (96·3%)	3657 (96·1%)
Mixed	18 (0·5%)	25 (0·7%)	25 (0·7%)
Asian	81 (2·1%)	67 (1·8%)	81 (2·1%)
Black	41 (1·0%)	29 (0·8%)	28 (0·7%)
Other	23 (0·6%)	19 (0·5%)	14 (0·4%)
Data missing	7 (0·2%)	13 (0·3%)	5 (0·1)
Living situation			
On my own	1043 (26·6%)	1017 (26·7%)	950 (25·0%)
With my spouse or partner	2701 (68·9%)	2635 (69·1%)	2684 (70·5%)
With a relative	145 (3·7%)	125 (3·3%)	150 (3·9%)
With a friend	18 (0·5%)	21 (0·6%)	15 (0·4%)
With a housemate	13 (0·3%)	15 (0·4%)	6 (0·2%)
Data missing	7 (0·2%)	13 (0·3%)	5 (0·1%)
Education			
GCSE or O-Levels	1433 (36·6%)	1274 (33·4%)	1273 (33·5%)
A-levels	677 (17·3%)	674 (17·7%)	655 (17·2%)
Diploma or BTEC	832 (21·2%)	846 (22·2%)	842 (22·1%)
Undergraduate	632 (16·1%)	701 (18·4%)	701 (18·4%)
Postgraduate	278 (7·1%)	264 (6·9%)	278 (7·3%)
PhD	68 (1·7%)	54 (1·4%)	56 (1·5%)
Data missing	7 (0·2%)	13 (0·3%)	5 (0·1%)
Employment			
Retired	2711 (71·2%)	2665 (69·7%)	2766 (70·4%)
Unemployed	125 (3·3%)	140 (3·7%)	144 (3·7%)
Full-time employed	375 (9·8%)	367 (9·6%)	377 (9·6%)
Part-time employed	310 (8·1%)	352 (9·2%)	311 (7·9%)
Unpaid work	119 (3·1%)	115 (3·0%)	131 (3·3%)
Unable to work	29 (0·8%)	43 (1·1%)	44 (1·1%)
Combination	141 (3·7%)	144 (3·8%)	154 (3·9%)
Data missing	7 (0·2%)	13 (0·3%)	5 (0·1%)
Comorbidities present	3040 (77·6%)	2964 (77·7%)	3001 (78·9%)
Data missing	7 (0·2%)	13 (0·3%)	5 (0·1%)
Number of comorbidities	2 (1–3)	2 (1–3)	2 (1–3)
Data missing	7 (0·2%)	13 (0·3%)	5 (0·1%)
Number of medications	3 (1–5)	3 (1–5)	3 (1–5)
Data missing	7 (0·2%)	13 (0·3)	5 (0·1%)
Family history of dementia	903 (25·5%)	846 (24·7%)	827 (24·2%)
Data missing	661 (17·3%)	668 (17·5%)	662 (16·9%)
Internet use			
Less often	59 (1·5%)	44 (1·2%)	56 (1·5%)
A few times each month	66 (1·7%)	54 (1·4%)	56 (1·5%)
A few times each week	641 (16·4%)	584 (15·3%)	563 (14·8%)
Every day	3154 (80·5%)	3131 (82·1%)	3130 (82·3%)
Data missing	7 (0·2%)	13 (0·3%)	5 (0·1%)
Brain activities			
Less than once a month	942 (24·0%)	939 (24·6%)	886 (23·3%)
Once a month	496 (12·7%)	457 (12·0%)	508 (13·4%)
Once every 2 weeks	123 (3·1%)	135 (3·5%)	122 (3·2%)
Once a week	302 (7·7%)	325 (8·5%)	342 (9·0%)
A few times a week	846 (21·6%)	788 (20·7%)	769 (20·2%)
Every day	1211 (30·9%)	1169 (30·7%)	1178 (31·0%)
Data missing	7 (0·2%)	13 (0·3%)	5 (0·1%)
Digit span test baseline	5·7 (3·2)	5·6 (3·1)	5·7 (3·1)
Data missing	7 (0·2%)	13 (0·3%)	5 (0·1%)
Paired associates baseline	3·4 (1·1)	3·4 (1·1)	3·4 (1·1)
Data missing	9 (0·2%)	14 (0·3%)	6 (0·1%)
Self-ordered search baseline	5·4 (3·3)	5·3 (3·4)	5·4 (3·3)
Data missing	8 (0·2%)	13 (0·3%)	5 (0·1%)
Baddeley verbal reasoning score baseline	15·3 (6·2)	15·1 (6·4)	15·1 (6·4)
Data missing	8 (0·2%)	13 (0·3%)	5 (0·1%)
Age-associated cognitive decline flag	3919 (100·0%)	3813 (100·0%)	3805 (100·0%)
Data missing	8 (0·2%)	13 (0·3%)	5 (0·1%)
Mild cognitive impairment flag	2441 (62·3%)	2359 (61·9%)	2390 (62·8%)
Data missing	8 (0·2%)	13 (0·3%)	5 (0·1%)
Informant Questionnaire on Cognitive Decline in the Elderly	3·1 (0·3)	3·1 (0·4)	3·1 (0·3)
Data missing	7 (0·2%)	13 (0·3%)	5 (0·1%)
Instrumental Activities of Daily Living	1·0 (2·5)	1·1 (2·7)	1·0 (2·5)
Data missing	10 (0·3%)	16 (0·3%)	6 (0·2%)
Short Warwick–Edinburgh Mental Wellbeing Scale	25·5 (4·7)	25·7 (4·8)	25·7 (4·7)
Data missing	7 (0·2%)	13 (0·3%)	5 (0·1%)
EQ-5D index	0·799 (0·190)	0·799 (0·193)	0·796 (0·190)
Data missing	7 (0·2%)	13 (0·3%)	5 (0·1%)
SF-12 PCS	41·8 (9·2)	41·9 (9·0)	41·9 (9·0)
Data missing	7 (0·2%)	13 (0·3%)	5 (0·1%)
SF-12 MCS	50·2 (9·2)	50·3 (9·1)	50·4 (9·0)
Data missing	7 (0·2%)	13 (0·3%)	5 (0·1%)
Geriatric Depression Scale			
Not depressed	1959 (50·0%)	1947 (51·1%)	1908 (50·1%)
Uncertain	1081 (27·6%)	1002 (26·3%)	1061 (27·9%)
Depressed	880 (22·4%)	864 (22·7%)	836 (22·0%)
Data missing	7 (0·2%)	13 (0·3%)	5 (0·1%)
IPAQ MET mins	3972 (1980–6278)	3899 (1977–6217)	3786 (2034–6132)
Data missing	292 (7·7%)	279 (7·3%)	262 (6·7)
Self-efficacy for exercise	53·3 (21·0)	53·4 (21·1)	53·7 (21·3)
Data missing	7 (0·2%)	13 (0·3%)	5 (0·1%)
Locus of Causation for Exercise	4·6 (1·5)	4·6 (1·5)	4·6 (1·6)
Data missing	7 (0·2%)	13 (0·3%)	5 (0·1%)
Social Support Survey	76·9 (26·3)	76·5 (26·4)	77·4 (25·7)
Data missing	7 (0·2%)	13 (0·3%)	5 (0·1%)
Food Frequency Questionnaire prudent diet score	0·1 (1·3)	0·1 (1·3)	0·2 (1·3)

Data are mean (SD), n, n (%), or median (IQR). Percentages for all columns were based on excluding missing cases. Comorbidities include heart problems, heart valve problems, back or neck pain, cancer, claudication, Crohn’s disease or colitis, depression or anxiety, diabetes, high blood pressure, irritable bowel syndrome, liver problems, lung problems, osteoarthritis, psychosis, renal problems, rheumatoid arthritis, and stroke or transient ischaemic attack. Brain activities included crosswords, word searches, or sudoku type activities. The digit span test assessed attention. The paired associates test assessed the verbal short-term memory. The self-ordered search assessed the spatial working memory. Age-associated cognitive decline is 1 SD below the norm for verbal reasoning. Mild cognitive impairment is 1·5 SD below the norm for verbal reasoning. Informant Questionnaire on Cognitive Decline in the Elderly scores are 1=much improved to 5=much worse. Instrumental Activities of Daily Living scores are 0 to 35 with lower scores indicating less help needed. Short Warwick–Edinburgh Mental Wellbeing Scale scores range from 7 to 35, and higher scores indicate higher positive mental wellbeing.

EQ-5D assessed the health-related quality of life. SF-12 ranged from 0 to 100, with higher scores indicating better health-related quality of life. Self-efficacy for exercise scores range from 0 to 90 with higher scores indicating greater higher self-efficacy for exercise. Locus of Causation for Exercise scores range from 1=strongly disagree to 7=strongly agree, of which high scores indicate greater self-determination. The Social Support Survey scores range from 0 to 100 with higher scores indicating greater support. SF-12=Short Form Health Survey Questionnaire. PCS=Physical Component Summary. MCS=Mental Component Summary. IPAQ=International Physical Activity Questionnaire. MET=minutes metabolic equivalent of time minutes.

Median time at which primary outcome was documented at 1 year was 366 days in all groups but with slightly varying IQR (370–402 for the Active Brains group; 370–400 for the Active Brains plus support group; 369–396 for the control group). The control group had substantially improved mean scores on the Baddeley reasoning test after 12 months (from 15·1 to 18·3, a standardised effect of 0·44). This is unlikely to be due to regression to the mean because SDs increased at follow-up in all groups: from 6·2 to 8·3 in the control group, from 6·4 to 8·7 in the Active Brains plus support group, and from 6·4 to 8·6 in the Active Brains group.

At 12 months, compared with the control group, mean Baddeley scores were higher in the Active Brains group (mean 18·7 adjusted mean difference compared with control +0·50, 95% CI 0·13–0·87) and the Active Brains plus support group (mean 19·2, +0·97; 0·59–1·34; table 2). These differences in scores reflect standardised effects of 0·06 and 0·11, respectively. This improvement was sufficient that a minority of all groups no longer met the criteria for AACD (883 [28·4%] of 3113 in the control group; 842 [32·8%] of 2566 in the Active Brains group; and 811 [31·6%] of 2546 in the Active Brains plus support group). There were very similar findings from the complete case analysis, and including a random effect for practice gave very similar results to those of the primary analysis (Active Brains: 0·52 [95% CI 0·13–0·90]; Active Brains plus support 0·98 [0·59–1·37]; intracluster correlation 0·004).

**Table 2:** Primary outcome of Baddeley verbal reasoning score at 1 year

	Active Brains group (n=3810)	Active Brains plus support group (n=3826)	Control group (n=3927)	Active Brains group *vs* control group, mean difference (95% CI)		Active Brains plus support group *vs* control group, mean difference (95% CI)	
				Unadjusted	Adjusted∗	Unadjusted	Adjusted∗
Multiple imputation analysis	18·7 (0·16), n=3810	19·2 (0·16), n=3826	18·3 (0·14), n=3927	0·38 (–0·04 to 0·79)	0·50 (0·13 to 0·87); p=0·008	0·87 (0·45 to 1·29)	0·97 (0·59 to 1·34); p<0·001
Complete cases analysis	19·0 (0·15), n=2564	19·5 (0·15), n=2566	18·5 (0·13), n=3113	0·54 (0·10 to 0·99)	0·51 (0·12 to 0·91)	1·01 (0·57 to 1·46)	0·97 (0·58 to 1·36)

Data are mean (SE), unless otherwise specified.

∗ Adjusted for baseline score, age, sex, index of multiple deprivation, number of comorbidities, and polypharmacy.

1919 (62%) of 3113 of the control group met the cognitive criteria for MCI at baseline, and by 12 months this had improved to 48% (1493 of 3113), consistent with the mean scores, but the Active Brains plus support group did slightly better at 44% (an 8% relative reduction compared with the control group; risk ratio 0·92 [95% CI 0·87–0·97]). Similarly in the control group, of those meeting the cognitive criteria for MCI at baseline, 329 (17·1%) of 1919 met the cognitive criteria for SCI at 12 months, and compared with the control group, there was a significant 18% reduction meeting the cognitive criteria for SCI in the Active Brains plus support group (0·82 [0·70–0·96]). Compared with the control group, by 12 months there were slightly fewer individuals in the Active Brains group who met the cognitive criteria for MCI (1196 [46·7%] of 2564) and cognitive criteria for SCI (242 [15·5%] of 1558), but neither difference was statistically significant compared with the control group. Other secondary outcomes are also reported (table 3).

**Table 3:** Secondary outcomes at 1 year

	Active Brains group (n=3810)	Active Brains plus group support (n=3826)	Control group (n=3927)	Active Brains group *vs* control group, adjusted∗ relative effect (95% CI)	Active Brains plus support group *vs* control group, adjusted∗ relative effect (95% CI)
Cognitive criteria for AACD at baseline with cognitive criteria for MCI at 1 year	1196/2564 (46·7%)	1130/2566 (44·0%)	1493/3113 (48·0%)	0·97 (0·92 to 1·02)	0·92 (0·87 to 0·97)
Cognitive criteria for MCI at baseline with cognitive criteria for SCI at 1 year	242/1558 (15·5%)	215/1531 (14·0%)	329/1919 (17·1%)	0·91 (0·78 to 1·05)	0·82 (0·70 to 0·96)
Digit span test	6·1 (2·7), n=2507	6·0 (2·7), n=2486	6·0 (2·9), n=3068	0·06 (–0·08 to 0·20)	0·00 (–0·14 to 0·14)
Paired associates test	3·6 (1·1), n=2512	3·6 (1·1), n=2514	3·5 (1·1), n=3074	0·04 (–0·01 to 0·10)	0·04 (–0·01 to 0·10)
Self-ordered search	5·7 (3·2), n=2429	5·9 (3·2), n=2424	5·7 (3·3), n=2977	0·03 (–0·14 to 0·20)	0·20 (0·03 to 0·37)
Informant Questionnaire on Cognitive Decline in the Elderly	3·0 (0·4), n=2533	3·0 (0·5), n=2529	3·1 (0·4), n=3082	–0·05 (–0·07 to –0·03)	–0·06 (–0·08 to –0·04)
Instrumental Activities of Daily Living	1·4 (4·6), n=2531	1·4 (4·8), n=2527	1·2 (4·3), n=3079	0·17 (–0·03 to 0·38)	0·09 (–0·11 to 0·29)
Short Warwick–Edinburgh Mental Wellbeing Scale	24·8 (4·5), n=2376	25·0 (4·7), n=2366	24·9 (4·6), n=2971	–0·07 (–0·26 to 0·11)	0·01 (–0·18 to 0·38)
EQ-5D index	0·799 (0·183), n=2527	0·799 (0·193), n=2526	0·797 (0·192), n=3081	0·0007 (–0·005 to 0·007)	–0·00004 (–0·006 to 0·006)
SF-12 PCS	41·8 (8·9), n=2353	41·8 (9·1), n=2337	41·6 (9·1), n=2950	0·14 (–0·18 to 0·47)	0·12 (–0·20 to 0·44)
SF-12 MCS	49·8 (8·9), n=2353	50·0 (9·1), n=2337	50·0 (8·9), n=2950	–0·35 (–0·70 to 0·003)	–0·07 (–0·43 to 0·28)
Geriatric Depression Scale				..	..
Not depressed	1270/2359 (53·8%)	1280/2347 (54·5%)	1594/2959 (53·9%)	1 (ref)	1 (ref)
Uncertain	636/2359 (27·0%)	594/2347 (25·3%)	788/2959 (26·6%)	1 (ref)	1 (ref)
Depressed	453/2359 (19·2%)	473/2347 (20·2%)	577/2959 (19·5%)	0·98 (0·88 to 1·10)	1·03 (0·93 to 1·15)
IPAQ total MET min per week	4110 (2148–6388), n=2251	4158 (2148–6449), n=2243	4079 (2141–6570), n=2850	1 (–152 to 149)	4 (–147 to 155)
Self-Efficacy for Exercise	53·7 (20·2), n=2298	54·1 (20·9), n=2302	54·0 (21·0), n=2910	−0·29 (–1·22 to 0·65)	−0·03 (–0·97 to 0·90)
Locus of Causation for Exercise	4·7 (1·6), n=2296	4·7 (1·5), n=2280	4·7 (1·5), n=2899	−0·05 (–0·12 to 0·01)	−0·01 (–0·08 to 0·06)
Food Frequency Questionnaire prudent diet score	0·0 (1·0), n=1675	0·0 (1·0), n=1667	−0·1 (1·0), n=2226	−0·01 (–0·24 to 0·22)	−0·09 (–0·31 to 0·13)
Patient Enablement Index	3·8 (0·8), n=2373	3·8 (0·8), n=2360	3·7 (0·9), n=2971	0·14 (0·10 to 0·18)	0·13 (0·09 to 0·17)
Dementia	7/2517 (0·3%)	10/2504 (0·4%)	14/3061 (0·5%)	..	..

Data are n/N (%); mean (SD), n; median (IQR), n; unless stated otherwise. Cognitive criteria for AACD (SD 1 below norm for verbal reasoning), mild cognitive impairment (SD 1·5 below norm for verbal reasoning), and severe cognitive impairment (SD 2 below norm for verbal reasoning) were used. The digit span test assessed attention. Paired associates test assessed the verbal short-term memory. The self-ordered search assessed the spatial working memory. Informant Questionnaire on Cognitive Decline in the Elderly scores range from 1=much improved to 5=much worse. Instrumental Activities of Daily Living scores range from 0 to 35 with lower scores indicating less help needed. Short Warwick–Edinburgh Mental Wellbeing Scale scores range from 7 to 35 and higher scores indicate higher positive mental wellbeing. EQ-5D assessed health-related quality of life. The Short Form Health Survey Questionnaire scores range from 0 to 100, with higher scores indicating better health-related quality of life. Self-efficacy for exercise scores range from 0 to 90 with higher scores indicating greater higher self-efficacy for exercise. Locus of Causation for Exercise scores range from 1=strongly disagree to 7=strongly agree, of which higher scores indicate greater self-determination. Food Frequency Questionnaire prudent pattern score standardised mean 0 (SD 1). Patient enablement index scores range from 1 to 7 with higher scores indicating greater enablement. AACD=age-associated cognitive decline. MCI=mild cognitive impairment. SCI=severe cognitive impairment. PCS=Physical Component Summary. MCS=Mental Component Summary. IPAQ=International Physical Activity Questionnaire. MET min=metabolic equivalent of time minutes. SF-12=Short Form Health Survey Questionnaire.

∗ Adjusted for baseline score, age, sex, index multiple deprivation, comorbidities, and polypharmacy; relative risk for binary outcomes, mean difference for continuous outcomes.

There were no significant interactions for any subgroup, and in particular there were similar estimates of effect for older age group, female participants, ethnic minorities, and those from areas of relatively higher deprivation (albeit for the Active Brains plus support group, there was a suggestion of a lower effect in the more deprived participants). The exploratory sensitivity analyses did not alter the inferences: using definitions of AACD and MCI that included other domains yielded small numbers, and thus were difficult to interpret; there was no evidence of a difference in effect due to the imputation assumptions or due to the order of verbal reasoning tasks. For the subgroup analysis, see the appendix (p 7).

According to the definition of adherence specified in the Statistical Analysis Plan, 1937 (50·9%) of 3805 participants were adherent to the intervention in the Active Brains group and 1970 (51·7%) of 3813 in the Active Brains plus support group. Within this subgroup of adherent participants, the Complier Average Causal Effects was 0·75 (95% CI 0·19–1·33) in the Active Brains group and 1·42 (95% CI 0·85–1·99) in the Active Brains plus support group, indicating a slightly larger effect of the Active Brains intervention on the primary outcome in both the Active Brains groups compared with the ITT analysis (0·75, compared with 0·5 in the ITT analysis) and the Active Brains plus support group (1·42, compared with 0·97 in the ITT analysis) among those with better adherence.

Serious adverse events occurred in 33 (0·87%) of 3810 in the Active Brains group, 32 (0·84%) of 3826 in the Active Brains plus support group, and 28 (0·71%) of 3927 in the control group. Deaths occurred in 20 (0·5%) in the Active Brains group, 17 (0·4%) in the Active Brains plus support group, and 19 (0·5%) in the control group (table 4). All adverse events and serious adverse events were determined to be unrelated to the study treatment. The risk ratio (95% CI) was 1·21 (0·74–2·01) for the Active Brains group versus the control group and 1·17 (0·71–1·94) for the Active Brains plus support group versus control group.

**Table 4:** Serious adverse events

	Active Brains group	Active Brains plus support group	Control group
Occurrence of at least one serious adverse event	33/3810 (0·87%)	32/3826 (0·84%)	28/3927 (0·71%)
Death	20	17	19
Falls or fractures	3	2	1
Cardiovascular	1	5	0
Neurological	1	1	0
Cancer	0	2	1
Joint replacement or surgery	2	1	3
Other	6	4	4

Data are n/N (%) or n, unless otherwise specified. Cardiovascular serious adverse events include myocardial Infarction, stroke, and transient ischaemic attack. Neurological serious adverse events include Alzheimer’s disease and Parkinson’s disease. Other serious adverse events include giant cell arthritis, chest infection, rheumatoid arthritis, osteoarthritis, diabetes, and COPD.

Practical problems engaging with the recommendations, including not having enough time, suitable opportunities, or forgetting, appeared to be the most common barrier with more than two-thirds of both groups indicating experience of at least one of these barriers (appendix p 15). Overall, there was very little difference in the proportions of Active Brains group and Active Brains plus support group accessing the various elements of Active Brains. Just over 80% of both intervention groups accessed the start of the Active Brains intervention content and most of these completed the crucial introductory section that was intended to motivate users by explaining the rationale for Active Brains and the intended benefits of the behaviours that it would provide support for, addressing some common concerns and providing brief stories about others’ successes. More than 60% of both intervention groups reported following recommendations from “Getting Active” and just over 50% for each of strength and balance, break from sitting, and healthy eating (appendix p 14). In terms of the Active Brains website usage data, these percentages were lower for each element of Active Brains (appendix p 14). There was little difference for self-reported physical activity or a prudent diet between groups (table 3). Similarly, there was little difference for most self-reported brain activities between groups, but all groups had substantially improved their engagement with brain activities every day, from 30% at baseline to more than 50% at follow-up (appendix p 10). Both intervention groups had slightly fewer participants who reported that they had never engaged with each brain stimulating activity. However, there was a substantial difference for brain health websites. These websites could include both the Active Brains brain training games (which were accessed by just of 50% in both intervention groups, appendix p 14) and other websites; approximately 20% more of the control group never accessed websites for brain health, and approximately 10% more of the control group never accessed other brain health programmes (appendix p 10).

In the support group, 1030 (27%) of 3815 took up the potential offer of support and 969 (25%) of 3815 had some interaction with their supporter, of whom 858 (88·5%) of 969 had at least one support contact (either by phone or email). 456 (47%) of 969 had only one support contact (either phone or email) and 531 (55%) of 969 had telephone support.

## Discussion

This study shows that providing a website with brief additional support for physical activity, diet, and brain training is likely to have a modest effect on cognitive functioning. The clinical relevance and potential effect was evidenced by the significant reduction in the proportion of individuals with cognitive criteria for MCI and the cognitive criteria SCI at 1 year in the Active Brains plus support group. The only substantial difference between groups was the increased access by both intervention groups to brain training websites and games. The interventions are low cost and so likely to be cost-effective, but a full economic evaluation will only be available following the 5-year follow-up.

The importance of the study is highlighted by the UNs’ recognition of cognitive impairment and dementia as major non-communicable disease,^19^ and although there are currently drugs (eg, donanemab and targeting amyloid) that provide a modest effect in the progression of cognitive impairment, they are unlikely to be cost-effective.^20^ This makes prevention of cognitive decline by scalable low-cost interventions a priority, fitting with the UK NHS 10-year plan that prioritises prevention and the WHO global action plan.^1^ This is one of the few trials of a robustly developed brief multidomain behaviour change intervention for cognitive impairment designed for primary care, and one of the very few brief interventions to report longer term follow-up. Regarding strengths and limitations, this is the largest trial to address the effect on cognition of brief support for physical activity, diet, and brain training. The complex intervention was developed robustly with the person-based approach focused on inclusion of the intended users at all stages of development. The study was a pragmatic open trial, providing the optimal comparator for meaningful estimates of effectiveness. A further strength of the study is that all interventions are, in principle, readily scalable, which increases the possibility of significant national impact; although, there would need to be a mechanism to provide the facilitator support and to update the website. Our study also has some limitations. Digital exclusion is a problem for some older individuals, but internet access is rising sharply in this group, and in 2023, 71% of the people older than 65 years had internet access (Age UK survey) and 63% had all the necessary skills to use the internet effectively (eg, using a mouse or changing a password). Although accessibility to the study and intervention might have been limited to those who do not have good digital literacy, the novel methodology of recruitment via the internet and central support is not only efficient but potentially more convenient for some participants, particularly for those with limited mobility, or facing difficulty travelling to appointments**,** potentially reducing barriers to participation by taking the research to the patient. The mean scores in the control group improved overall, which probably suggests a motivated sample. This change could, in part, be regression to the mean, but no such effect was found in similar cohorts with repeated testing over several weeks^14^^,^^21^ or in previous trials,^8^ and SD increase in all groups at follow-up, which suggests regression to the mean is unlikely to be a major issue and cannot explain the significant change in behaviour in the control group. Cognitive decline might have contributed to attrition since baseline scores did predict missingness, but controlling for baseline cognitive scores did not materially alter the estimates. Although we made no allowance for multiplicity, the level of significance and number, pattern and consistency of both primary and key secondary outcomes (eg, enablement or IQCODE) that were significant across intervention groups make chance a very unlikely explanation. We did not power the study to be able to detect differences between the intervention groups, which would have made the study unfeasibly large. We recruited a relatively health-conscious group, indicated by their high baseline IPAQ-E scores, which could have increased the effect of the intervention. Conversely this meant there were ceiling effects, and there was no difference in reported physical activity or diet at follow-up, which makes it more likely that we have underestimated the effect of the multidomain intervention. Furthermore, the title of the study (Active Brains); cognitive assessment; questionnaires about diet, physical activity, and brain stimulating activities; evidence-based advice; and repeated cognitive testing and questionnaires (none of which are currently given routinely given in primary care) are likely to be prompts to behaviour change for participants, even in the control group. Of note is the finding that more than 50% of individuals report brain stimulating activities daily at follow-up versus 30% at baseline in all groups, further limiting our ability to detect between group differences. Invitations via cold calling provide relatively low uptake rates, but the PRIMIT trial (using similar methods) showed behavioural intentions that were very similar subsequently outside the trial context.^22^

Considering generalisability, we only have data for 5% (of invitees) on reasons for non-participation, but lack of access to internet (1344 [37%]) was the near equal most common reason with not interested in participating (1256 [34%]), which suggests lack of access was not the major factor affecting the generalisability of the results. We recruited widely from a range of communities (including from deprived areas and areas with high ethnic minority population), and the sample had similar numbers of ethnic minority communities (5%) compared to 5% predicted in this age group from the 2021 census, and similar educational levels to census data. Unmeasured confounding cannot be rule out, but there was no clear evidence of a differential effect of age, sex or gender, ethnic minority status, or deprivation (albeit a possible suggestion of lesser effect in the Active Brains plus support group) which suggests these results are likely to be generalisable.

Putting the study findings in the context of previous literature, the multidomain intensive intervention in the FINGER trial showed modest improvements in cognition in both intervention and control groups with small between group differences (Z score of 0·22 between groups), with similar results from the US POINTER trial.^6^ As with the current trial, the FINGER trial found increased odds of progression of cognitive impairment in the control group. FINGER was very much more intensive than the Active Brains approach, with several individual and group sessions for diet, physical activity, cognitive training, and regular additional reviews by clinicians (nurse and physician several times in the year). The US POINTER trial did include a group having briefer support, but included six support meetings over 2 years and annual clinical reviews with weight and blood pressure measurements and test results (eg, lipids). The Maintain Your Brain trial^10^ intervention provided similar results over 3 years to the FINGER trial, and was more scalable (with user generated contact with supervisors, and supervisor feedback for poorer performing participants); however, the study did not recruit from primary care but from the 45 and Up Study, an existing community cohort study (ie, already a research-interested population).

The study does raise implications for research, patient care, and policy. The encouraging 1-year results on cognitive scores suggests that research to document the 5-year effect remains important, and given the relatively healthy population we included, research is also needed for a wider range of participants. For patient care these results suggest that providing evidence-based advice about protecting cognitive health is likely to be helpful. However, cognitive testing is not currently available in routine practice, even though patients could join the PROTECT cohort where both testing and braining training games are available. Combined with the results of other multidomain intervention this suggests that the policy agenda should consider focussing on how to provide brief multidomain interventions more widely.

## Data sharing

Individual participant data that underlie the results reported in this Article after de-identification will be made available to achieve aims proposed by researchers who provide a methodologically sound proposal, on request to PLi or LY.

## Declaration of interests

AC declares research grants from DailyColors and consultancy fees from Suven, Signant, and Sunovion. BG declares support for attending meetings or travel from the University of Southampton. GY declares grants from the National Institute for Health and Care Research (NIHR) and the British Heart Foundation (paid to the University of Leicester) and being a member of the NIHR programme grant for Applied Research Committee and NIHR Cross Network Multiple Long Term Conditions) working group. HB declares being director of product at Signant Health; former employment at University of Exeter, with several projects ongoing to date; and grants and consulting fees from Innovate. JG declares grants from the Medical Research Council (MRC) Dementias Platform UK, MRC Motor Neurone Disease (MND) Translation Accelerator and MRC/NIHR MND Translation catalyst; payment to visit Dementias Platform Korea from Yonsei University (Seoul, South Korea); and membership of the Cohortes France Advisory Board and UK Secure e Research Platform Advisory Board. JO'B declares grants from the Cambridge NIHR Biomedical Research Centre; consulting fees from Biogen, Roche, GE Healthcare, and Okwin; receiving honorarium for lectures from GE Healthcare; participation on data safety and monitoring boards for TauRx and Novo Nordisk; being chairman of the UK Alzheimer's Society Research Strategy Council; and receiving grants, paid to his institution, chemical precursors and scanning time from Avid/Lilly, Merck, UCB, and Alliance Medical. SRa declares royalties paid by Wiley Blackwell, Springer, Altman Publishing, and Cambridge University Press; owning a private medical practice and medicolegal work; receiving payment for a presentation from Brorson & Sande; support for attending a meeting from Takeda; and being director of a PLC Metaphaura. SG declares receiving honoraria for contributions to postgraduate educational meetings from AstraZeneca. TK reports that his employer, University of Southampton, received funding for time spent on the study. All other authors declare no competing interests.
