# Supplementary material for: A digital intervention to protect cognitive health in older adults with cognitive impairment: a three-arm, open-label, randomised controlled trial
Source: Lancet Prim Care. 2026 Aug;2(8):None. doi: 10.1016/j.lanprc.2026.100192 (PMC13552317; doi:10.1016/j.lanprc.2026.100192)
Supplement: Supplementary file 1 [file mmc1.pdf]

THE LANCET
Primary Care

Supplementary appendix

This appendix formed part of the original submission and has been peer reviewed. We post it as supplied by the authors.

Supplement to: Little P, Essery R, Somerville J, et al. A digital intervention to protect cognitive health in older adults with cognitive impairment: a three-arm, open-label, randomised controlled trial. *Lancet Prim Care* 2026. https://doi.org/10.1016/j.lanprc.2026.100192

Appendix

Table of contents

**Item**	**Page**
Baddeley test	**2**
Active Brain Intervention	**2**
Table 1. subgroups analysis for verbal reasoning score	**6**
Table 2. Sensitivity Analyses	**8**
Table 3. Self reported Brain activities	**9**
Reasons for not taking part	**12**
Table 4. Website usage measures	**13**
Table 5. Self reported process measures	**14**
Table 6. Baseline characteristics by missingness	**15**
Table 7. Protocol changes	**17**
Sample size calculation	**18**
Patient and Public involvement	**18**
References	**19**

Baddeley test

This was originally developed as a short test of executive function/fluid intelligence test[1], and is a very well validated test of executive function [2], detecting consistent cognitive decline over two years, correlating strongly with the gold standard Raven Progressive Matrices test[3] and with decline in function, and with no evidence of learning effects[2,4,5]. Computerized neuropsychological testing is in widespread usage including CANTAB, COGSTATE and the PROTECT cognitive test systems – validated over 50,000 users and 2.5million assessments [2,6-8] and has the major advantage of avoiding inter-rater reliability bias.

Active brains intervention

The Active Brains digital intervention provides interactive, tailored support to encourage older adults to engage in behaviours beneficial for their cognitive and physical health. The intervention content utilises behaviour change techniques to motivate users to engage in, and maintain, evidence-based activities for sustaining cognitive health in later life. All intervention content has been iteratively developed with extensive input from the target user group to ensure that it is highly accessible and engaging. For the first seven months, users will have access to the Active Brains 'Starter Section' which provides support for users to initiate changes to their lifestyle in line with the intervention's recommendations.

Active Brains Starter Section

Within the first section of Active Brains, three primary modules become available to users sequentially. Within these modules, users have access to: information addressing common concerns, instruction about recommended activities, facilities to set and review goals about their chosen activities, tailored motivational feedback about their progress, and reminder emails to motivate them to continue with their activities and to revisit intervention content as appropriate. Additional detail about each of the main modules is provided below.

Active Lives

The 'Active Lives' module supports users in gradually increasing their levels of physical activity in a manner that is most suited to their present physical capabilities and level of activity. Within this module, there are three sub-modules that users are directed to differentially depending on which is deemed to be most beneficial for them. The 'Getting Active' sub-module provides support and ideas about activities to try to increase individuals' overall levels of physical activity. The 'Breaks from Sitting' sub-module supports individuals in reducing sedentary behaviour by suggesting simple changes that can be made to daily routines. The 'Strength and Balance Training' module provides video demonstrations of simple strength and balance exercises and suggestions about how these can be built into daily activities. The level of exercise difficulty is tailored to the user and their progress. Whilst tailored recommendations are made about which sub-module users may find most helpful, they have access to all three sub-modules.

Brain Training

The Brain Training module follows on from Active Lives and is accessible to participants after four weeks. This module provides brief information about the rationale and evidence base for Brain Training tasks and how they are intended to work. The module initially provides participants with access to six online brain training games (via the existing PROTECT-UK website) which they are encouraged to play between three and five times per week. Users are encouraged to set and review goals relating to their use of the brain training games. At regular intervals throughout the six month period of the starter session, six additional games are made available to users.[7] The games are described below:

Game Name	Description
**Crates**	Crates is a multi-level brain training game with two grids. The first grid starts completely filled with interlocking-coloured shapes, while the second grid shows a silhouette of the same shapes but with some of the shapes removed. The aim of the game is for the user to remove shapes in the first grid until it matches the silhouette in the second grid. If a shape is removed and the shape above it is no longer supported, the unsupported shape falls to the bottom of the grid. Each level is slightly more difficult than the previous one, with the user needing to remove more shapes and allow more shapes to fall into position to match the silhouette.
**Loop the loop**	Loop the Loop is a multi-level brain training game with a single grid of cells with clearly defined vertices and edges. The grid initially starts three cells wide and three cells high, with the edges of the cells unselected. Some of the cells contain a number from 0 to 3 and the rest are empty.<br>The aim of the game is for the user to draw a single path, that does not cross itself or include branches, by selecting the edges of cells in the grid. The number of selected edges for a cell must match the number inside. An empty cell can have any number of selected edges. The path the user creates must join up with itself to create a single loop. When the user completes a loop, they "submit" their solution. If the solution is incorrect the user is asked to try again and to unselect and select different edges to create a different loop. If the solution is correct they progress to the next level which is more difficult.
**Odd One Out**	Odd One Out is a multi-level brain training game where an odd number of shapes are shown to the user and they are asked to select the shape that does not match any of the others. The grid initially starts with three flower shapes made from a circle (pistil) that is surrounded by coloured shapes, representing petals. These shapes can vary in number, colour and shape between flowers but are the same for any given flower. The centre of the flower (pistil) contains a shape that can vary in shape and colour between flowers. The aim of the game is for the user to find pairs of flowers by comparing the number, shape and colours of the petals, as well as the shape and colour of the shape inside the circle (pistil). Flowers are considered a pair if they have more matching features than any of the other flowers. The user must then select the odd one out; the flower that is not part of a pair. A correct response moves the user to the next level which is more difficult.
**See Saws**	See Saws is a multi-level brain training game where multiple see saws are drawn as viewed from the side, with one or more coloured shapes on each end. One of each of the different shapes on the see saws are shown below the see saws for the user to select to indicate which shape is the heaviest. The aim of the game is for the user to calculate, based on how the see saws are tipped, and the number of each coloured shape on the end of each see saw, which coloured shape is the heaviest. A correct response moves the user to the next level which is more difficult.
**Slider**	Slider is a multi-level brain training game where a grid of cells is shown to the user. All the cells contain a unique number from 1 to one less than the number of cells, with one of the cells left empty. The number cells are scrambled so they are not in order. The game starts

	with a three-by-three grid, containing the numbers 1 to 8. The numbers are out of order and the grid contains one empty cell. The aim of the game is for the user to select a numbered cell next to the empty cell. The numbered cell then slides into the empty cell, allowing the user to re-arrange the numbers so that they are in numerical order (when reading left to right across the grid from the top row, wrapping to the left-most cell on the next row) with the empty cell in the bottom right corner of the grid. A correct solution moves the user to the next level which is more difficult.
**Tower of London**	Tower of London is a multi-level brain training game where two sets of vertical columns are shown to the user. The first set of columns contains a variety of coloured shapes stacked one upon another. Some columns may be filled to the top but other columns have room for more or are empty. The second set of columns contains the same set of coloured shapes in a fixed arrangement. The aim of the game is for the user to rearrange the coloured shapes in the first set of columns until they match the arrangement of coloured shapes in the second set of columns. A correct solution moves the user to the next level which is more difficult.

Eat for Health

The Eat for Health module follows on from Brain Training and becomes accessible to participants eight weeks after they first use the intervention. This module outlines the benefits of healthy eating for cognitive and physical health. It offers suggestions about specific foods and food groups that are beneficial for cognitive health. It also provides users with information and techniques to allow them to make healthy changes to their existing eating behaviours more generally (e.g. increasing fruit and vegetable consumption, cutting down on processed foods).

Active Brains Booster Section

After seven months, it was expected that the primary needs of intervention users would be somewhat different - that, with support from the Active Brains Starter Sessions, the recommended activities that users had chosen to engage with would have become more habitual behaviours. These users were, therefore, expected to have less need to rely on the intervention content so extensively. Accordingly, at this stage, users were directed to access the Active Brains Booster section. This content included access to all content from the Starter Section, as well as advice on a 'maintenance' schedule for brain training, and centralised links to external resources for additional support and to extend their progress with the behavioural changes made.

Our previous qualitative studies (REC reference SC/17/0463), explored the acceptability of Active Brains amongst target users. Analysis of our think aloud and semi-structured interviews (n=59) informed iterative changes to the intervention content throughout development and indicated that the intervention is acceptable to older adults with and without cognitive decline. This phase also sought extensive input from PPI representatives in the development of intervention content and participant recruitment materials. Our development phase qualitative work is currently being prepared for submission for publication.

Central Facilitator Support

Central facilitators were either research nurses based in the participating practices or research networks, or University employees. All were provided with manualised training materials and offered the opportunity to attend a training session with a member of the research team (RE). Patients in the group receiving support from a central

facilitator (in addition to the website) were offered a brief (10 minute) telephone support call 2 weeks after they begin the study. In this support call they discussed the cognitive/lifestyle changes that they chose to try. Patients were offered two more support contacts by phone (up to 10 minutes) or email to support them in making behavioural changes. If the participant had not accessed Active Brains at the time of contact, the supporter checked whether the participant had technical difficulties and/or talked through any other barriers to getting started.

For patients who felt the need, up to 7 further email or phone contacts could be arranged. In the feasibility study only one participant requested additional contact beyond the three initial calls (n = 120 in support arm). In our previous studies only around 10-15% of patients have required this further support.

The central facilitator used the CARE (Congratulate, Ask, Reassure, Encourage) approach to provide support to patients, which is based on Self-Determination Theory, designed to promote autonomous motivation for making behavioural changes – and used successfully in our previous trials and has been shown to be acceptable to patients and staff supporting patients {Smith, 2017 #4}. Supporters provided brief technical website support to all participants as required throughout all contact points (e.g. advice on logging in problems). To maximise fidelity of support delivery, a 'checklist' of the CARE approach was provided in the supporter training materials that supporters were encouraged to use to guide their calls with participants. This also included space to write notes about these interactions. As a means of fidelity check, supporters were encouraged to share the completed checklists/notes from one or two of these anonymous interactions with the research team. A member of the research team with expertise in the CARE approach provided feedback to the supporter as appropriate.

Table 1. Subgroup analysis for verbal reasoning score

			Active Brains vs Control		Active Brains + Support vs Control	
			Mean difference in verbal reasoning score within subgroup (95% CI)	Interaction term – mean difference between subgroups (95% CI)	Mean difference in verbal reasoning score within subgroup (95% CI	Interaction term – mean difference between subgroups (95% CI
cMCI						
	Yes	7190	0.46 (-0.06, 0.97)	-0.13 (-0.93, 0.67)	1.02 (0.50, 1.54)	0.15 (-0.65, 0.95)
	No	4347	0.58 (-0.01, 1.18)	REF	0.88 (0.29, 1.47)	REF
Age						
	75 or over	3629	-0.08 (-0.76, 0.59)	-0.82 (-1.67, 0.03)	0.76 (0.08, 1.43)	-0.32 (-1.17, 0.52)
	Under 75	7909	0.76 (0.28, 1.23)	REF	1.07 (0.60, 1.55)	REF
Gender[1]						
	Female	6672	0.44 (-0.07, 0.95)	-0.19 (-0.99, 0.61)	1.02 (0.50, 1.55)	0.10 (-0.69, 0.89)
	Male	4866	0.64 (0.03, 1.24)	REF	0.91 (0.32, 1.50)	REF
Deprivation						
	Higher deprivation (deciles 1-5)	3292	0.64 (-0.12, 1.40)	0.17 (-0.70, 1.04)	0.40 (-0.35, 1.15)	-0.81 (-1.68, 0.07)
	Lower deprivation	8191	0.46 (0.01, 0.92)	REF	1.20 (0.74, 1.65)	REF
Comorbidities						
	2 or more	5833	0.58 (0.03, 1.13)	0.13 (-0.65, 0.91)	0.87 (0.32, 1.42)	-0.21 (-0.99, 0.57)
	Less than 2	5705	0.46 (-0.09, 1.02)	REF	1.08 (0.53, 1.63)	REF
Polypharmacy						
	5 or more	3096	0.34 (-0.41, 1.09)	-0.26 (-1.16, 0.63)	0.49 (-0.28, 1.25)	-0.67 (-1.58, 0.23)
	4 or fewer	8442	0.58 (0.13, 1.04)	REF	1.14 (0.68. 1.59)	REF
Healthy diet						
	Healthier[1] (upper quartile)		-2.03 (-4.63, 0.58)	-2.33 (-5.13, 0.48)	0.44 (-2.15, 3.04)	0.20 (-2.60, 3.00)
	Less healthy		0.36 (-0.96, 1.67)	REF	0.25 (-1.07, 1.58)	REF
Physical activity						
	Higher[1] (upper quartile)		0.63 (0.16, 1.10)	0.41 (-0.45, 1.26)	0.97 (0.50, 1.43)	-0.01 (-0.87, 0.86)
	Lower		0.22 (-0.49, 0.94)	REF	0.98 (0.26, 1.67)	REF
Depression (GDS)						
	Depressed	1503	0.84 (-0.12, 1.80)	0.27 (-0.79, 1.32)	0.66 (-0.30, 1.62)	-0.46 (-1.51, 0.59)
	Not depressed	6162	0.58 (0.12, 1.04)	REF	1.17 (0.71, 1.63)	REF
Retired						
	Retired	8142	0.39 (-0.07, 0.84)	-0.43 (-1.30, 0.44)	0.93 (0.47, 1.39)	-0.14 (-1.01, 0.73)
	Not retired	3421	0.82 (0.06, 1.57)	REF	1.05 (0.31, 1.80)	REF

Ethnicity[1]						
	All other ethnicities	451	1.08 (-0.98, 3.14)	0.57 (-1.47, 2.61)	0.92 (-1.24, 3.09)	0.35 (-1.76, 2.46)
	White	11087	0.50 (0.10, 0.89)	REF	0.96 (0.56, 1.36)	REF
Living alone[1]						
	Yes	3010	0.27 (-0.48, 1.03)	-0.28 (-1.18, 0.62)	0.56 (-0.17, 1.30)	-0.58 (-1.47, 0.31)
	No	8528	0.58 (0.11, 1.03)	REF	1.12 (0.66, 1.58)	REF

1 Post-hoc analysis; Interaction term - difference in intervention effect between subgroups

Table 2. Sensitivity analyses

		Active Brains N=3810		Active Brains + Support N=3826		Control N=3927	Active Brains vs Control Relative effect (95% CI)	Active Brains + Support vs Control Relative effect (95% CI)
**AACD/MCI using stricter definitions (1 SD below norm for IADL and verbal reasoning)**								
AACD at 1 year according to stricter definition[1], n (%) RR	2699	204 (7.6%)	2703	206 (7.6%)	3257	197 (6.1%)	**1.25 (1.03, 1.50)**	**1.25 (1.04, 1.51)**
MCI at 1 year according to stricter definition[2], n (%) RR	2481	424 (17.1%)	2479	381 (15.4%)	3036	519 (17.1%)	1.00 (0.89, 1.12)	0.90 (0.77, 1.01)
**Baddeley verbal reasoning score at 1 year (delta-based multiple imputation)**								
δ=+0.15 standardised pts , mean (SE)		19.1 (0.16)		19.6 (0.16)		18.6 (0.14)	0.65 (0.28, 1.02)	1.12 (0.75, 1.50)
δ=-0.15 standardised pts, mean (SE)		18.3 (0.16)		18.8 (0.16)		18.0 (0.14)	0.34 (-0.03, 0.72)	0.81 (0.43, 1.19)
**Baddeley verbal reasoning score at 1 year by order of verbal reasoning tests**								
Third task	1027	18.9 (8.9)	1048	19.8 (8.6)	1289	18.5 (8.4)	0.51 (-0.10, 1.12)	1.09 (0.48, 1.70)
First task	1537	19.1 (8.4)	1518	19.3 (8.8)	1824	18.5 (8.3)	0.54 (0.03, 1.04)	0.90 (0.39, 1.40)

[1]AACD strict flag: 1SD below norm for verbal reasoning AND 1SD below norm for IADL (i.e., >7.18 on IADL)

[2]MCI strict flag: 1.5SD below the norm for verbal reasoning AND 1.5SD below the norm for self-ordered search

Relative risk for binary outcomes, mean difference for continuous outcome.

Table 3. Self reported brain activities

	Active Brains N=3810	Active Brains + Support N=3826	Control N=3927
Crosswords			
Never	347 (15.4%)	332 (14.7%)	474 (16.4%)
Once or twice	220 (9.8%)	241 (10.7%)	281 (9.7%)
Once or twice per month	205 (9.1%)	202 (8.9%)	237 (8.2%)
Once or twice per week	442 (19.7%)	429 (19.0%)	547 (18.9%)
Every day	1033 (46.0%)	1056 (46.7%)	1355 (46.8%)
Reading newspapers			
Never	399 (17.7%)	386 (17.1%)	518 (17.9%)
Once or twice	207 (9.2%)	205 (9.1%)	290 (10.0%)
Once or twice per month	152 (6.8%)	137 (6.1%)	195 (6.7%)
Once or twice per week	551 (24.5%)	568 (25.2%)	706 (24.4%)
Every day	939 (41.8%)	962 (42.6%)	1183 (40.9%)
Reading books			
Never	218 (9.7%)	205 (9.1%)	303 (10.4%)
Once or twice	286 (12.7%)	283 (12.5%)	394 (13.6%)
Once or twice per month	252 (11.2%)	255 (11.3%)	319 (11.0%)
Once or twice per week	381 (16.9%)	385 (17.1%)	447 (15.4%)
Every day	1118 (49.6%)	1130 (50.0%)	1437 (49.6%)
Playing boardgames/cards			
Never	800 (35.5%)	821 (36.4%)	1175 (40.6%)
Once or twice	654 (29.1%)	628 (27.8%)	746 (25.8%)
Once or twice per month	410 (18.2%)	391 (17.3%)	459 (15.9%)
Once or twice per week	232 (10.3%)	240 (10.6%)	305 (10.5%)
Every day	155 (6.9%)	178 (7.9%)	209 (7.2%)
Visiting museums			
Never	972 (43.2%)	971 (43.0%)	1319 (45.6%)
Once or twice	1003 (44.6%)	989 (43.8%)	1249 (43.2%)
Once or twice per month	253 (11.2%)	267 (11.8%)	295 (10.2%)
Once or twice per week	16 (0.7%)	24 (1.1%)	23 (0.8%)
Every day	5 (0.2%)	6 (0.3%)	5 (0.2%)
New hobbies, course, skill			
Never	1371 (61.2%)	1448 (64.1%)	1880 (65.0%)
Once or twice	556 (24.8%)	518 (22.9%)	673 (23.3%)
Once or twice per month	147 (6.6%)	115 (5.1%)	150 (5.2%)
Once or twice per week	105 (4.7%)	118 (5.2%)	123 (4.3%)
Every day	63 (2.8%)	59 (2.6%)	67 (2.3%)
Crafts			
Never	1094 (48.6%)	1125 (49.7%)	1506 (52.0%)
Once or twice	317 (14.1%)	324 (14.3%)	382 (13.2%)
Once or twice per month	264 (11.7%)	246 (10.9%)	272 (9.4%)
Once or twice per week	317 (14.1%)	301 (13.3%)	397 (13.7%)
Every day	259 (11.5%)	266 (11.8%)	338 (11.7%)
Art			
Never	1629 (72.7%)	1689 (74.9%)	2231 (77.1%)
Once or twice	292 (13.0%)	264 (11.7%)	296 (10.2%)
Once or twice per month	154 (6.9%)	131 (5.8%)	170 (5.9%)
Once or twice per week	129 (5.8%)	127 (5.6%)	147 (5.1%)
Every day	38 (1.7%)	44 (2.0%)	49 (1.7%)

Social club			
Never	1273 (56.8%)	1314 (58.3%)	1687 (58.3%)
Once or twice	212 (9.5%)	208 (9.2%)	237 (8.2%)
Once or twice per month	372 (16.6%)	352 (15.6%)	433 (15.0%)
Once or twice per week	376 (16.8%)	376 (16.7%)	527 (18.2%)
Every day	9 (0.4%)	4 (0.2%)	8 (0.3%)
Visiting friends			
Never	131 (5.8%)	148 (6.6%)	141 (4.9%)
Once or twice	356 (15.8%)	344 (15.2%)	394 (13.6%)
Once or twice per month	787 (35.0%)	834 (37.0%)	992 (34.3%)
Once or twice per week	917 (40.8%)	884 (39.2%)	1291 (44.6%)
Every day	57 (2.5%)	46 (2.0%)	76 (2.6%)
Listening to music			
Never	126 (5.6%)	112 (5.0%)	168 (5.8%)
Once or twice	218 (9.7%)	177 (7.8%)	243 (8.4%)
Once or twice per month	234 (10.4%)	209 (9.3%)	270 (9.3%)
Once or twice per week	561 (25.0%)	574 (25.5%)	767 (26.5%)
Every day	1106 (49.3%)	1183 (52.5%)	1445 (49.9%)
DIY/home repairs			
Never	420 (18.7%)	399 (17.7%)	608 (21.0%)
Once or twice	534 (23.8%)	536 (23.8%)	694 (24.0%)
Once or twice per month	630 (28.0%)	643 (28.5%)	767 (26.5%)
Once or twice per week	522 (23.2%)	499 (22.1%)	650 (22.5%)
Every day	141 (6.3%)	176 (7.8%)	176 (6.1%)
Movies, restaurant, theatre, concert			
Never	278 (12.4%)	288 (12.7%)	397 (13.7%)
Once or twice	557 (24.8%)	584 (25.9%)	738 (25.5%)
Once or twice per month	1052 (46.8%)	1014 (44.9%)	1273 (43.9%)
Once or twice per week	354 (15.7%)	363 (16.1%)	480 (16.6%)
Every day	7 (0.3%)	10 (0.4%)	11 (0.4%)
Walking			
Never	169 (7.5%)	192 (8.5%)	307 (10.6%)
Once or twice	301 (13.4%)	263 (11.6%)	407 (14.0%)
Once or twice per month	418 (18.6%)	397 (17.6%)	512 (17.7%)
Once or twice per week	760 (33.7%)	818 (36.2%)	927 (32.0%)
Every day	604 (26.8%)	591 (26.1%)	744 (25.7%)
Gardening			
Never	227 (10.2%)	227 (10.2%)	370 (13.0%)
Once or twice	224 (10.1%)	220 (9.9%)	292 (10.2%)
Once or twice per month	489 (22.0%)	478 (21.5%)	534 (18.7%)
Once or twice per week	993 (44.6%)	1008 (45.3%)	1268 (44.5%)
Every day	291 (13.1%)	291 (13.1%)	385 (13.5%)
Group discussion			
Never	1013 (45.2%)	1062 (47.1%)	1543 (53.4%)
Once or twice	408 (18.2%)	404 (17.9%)	458 (15.8%)
Once or twice per month	417 (18.6%)	405 (18.0%)	468 (16.2%)
Once or twice per week	347 (15.5%)	335 (14.9%)	367 (12.7%)
Every day	55 (2.5%)	48 (2.1%)	53 (1.8%)
Charity/volunteer work			
Never	1350 (60.4%)	1392 (61.9%)	1873 (64.7%)
Once or twice	206 (9.2%)	245 (10.9%)	281 (9.7%)
Once or twice per month	262 (11.7%)	223 (9.9%)	265 (9.2%)
Once or twice per week	354 (15.8%)	342 (15.2%)	401 (13.9%)
Every day	64 (2.9%)	47 (2.1%)	72 (2.5%)

Playing a musical instrument			
Never	1969 (87.9%)	1968 (87.2%)	2552 (88.1%)
Once or twice	78 (3.5%)	75 (3.3%)	99 (3.4%)
Once or twice per month	49 (2.2%)	45 (2.0%)	65 (2.2%)
Once or twice per week	84 (3.7%)	93 (4.1%)	112 (3.9%)
Every day	61 (2.7%)	75 (3.3%)	67 (2.3%)
Cooking			
Never	108 (4.8%)	130 (5.8%)	200 (6.9%)
Once or twice	139 (6.2%)	157 (7.0%)	187 (6.5%)
Once or twice per month	142 (6.3%)	122 (5.4%)	141 (4.9%)
Once or twice per week	372 (16.6%)	403 (17.9%)	432 (14.9%)
Every day	1485 (66.1%)	1445 (64.0%)	1934 (66.8%)
Dancing			
Never	1763 (78.8%)	1810 (80.3%)	2325 (80.3%)
Once or twice	265 (11.8%)	221 (9.8%)	320 (11.1%)
Once or twice per month	76 (3.4%)	85 (3.8%)	86 (3.0%)
Once or twice per week	125 (5.6%)	131 (5.8%)	156 (5.4%)
Every day	8 (0.4%)	6 (0.3%)	6 (0.2%)
Website for brain health			
Never	752 (33.5%)	778 (34.5%)	1631 (56.4%)
Once or twice	461 (20.5%)	430 (19.1%)	412 (14.2%)
Once or twice per month	327 (14.6%)	316 (14.0%)	157 (5.4%)
Once or twice per week	346 (15.4%)	349 (15.5%)	262 (9.1%)
Every day	359 (16.0%)	384 (17.0%)	431 (14.9%)
Other programme for brain health			
Never	1663 (74.2%)	1743 (77.3%)	2474 (85.4%)
Once or twice	207 (9.2%)	170 (7.5%)	159 (5.5%)
Once or twice per month	111 (5.0%)	82 (3.6%)	64 (2.2%)
Once or twice per week	126 (5.6%)	117 (5.2%)	85 (2.9%)
Every day	134 (6.0%)	143 (6.3%)	114 (3.9%)

Reasons for not taking part

A sample (5.8%) of the patients who were sent a study invite were asked to return an opt-out slip indicating the reasons why they did not choose to take part in the study (n= 17144 / 292,435)

N=3656 (21%) of these were returned, indicating the following reasons for not taking part:

No Internet / Email /computer	1344
Not interested in study	1256
Not enough time to take part	526
Health Issues	343
Other	187

Table 4. website usage measures

**<u>Usage within first year</u>**	**Active Brains (n = 3810)**					**Active Brains + Support (n=3826)**				
Any access at all to AB (beyond study procedures)	83.3% (3169/3805)					81.0% (3087/3813)				
Completed intro pages about Active Brains before module content[1]	78.3% (2979/3805)					76.1% (2903/3813)				
Proportion accessing **start of** Active Lives	70.2% (2670/3805)					68.3% (2604/3813)				
Proportion accessing **start of** Getting Active (GA)	45.2% (1719/3805)					44.2% (1686/3813)				
Proportion accessing **start of** Strength and Balance (S+B)	43.1% (1639/3805)					43.0% (1637/3813)				
Proportion accessing **start of** Breaks from Sitting (BfS)	28.0% (1064/3805)					28.4% (1081/3813)				
Worked through any one of three PA sub-modules at least once[2]	48.8% (1856/3805)					48.2% (1839/3813)				
Proportion accessing BT games	50.5% (1921/3805)					50.4% (1920/3813)				
Proportion accessing start of Eat for Health	40.6% (1543/3805)					41.5% (1583/3813)				
	**Mean**	***(SD)***	**Median**	**Range**		**Mean**	***(SD)***	**Median**	**Range**	
				**Min**	**Max**				**Min**	**Max**
Average number of BT sessions[3] (inclusive of all)	13.37	*37.57*	1.00	0	463	15.67	*46.55*	1.00	0	862
Average number of BT sessions[3] (of those who accessed)	26.46	*49.48*	6.00	1	463	31.13	*61.83*	8.00	1	862

Table 5. Self reported process measures in intervention arms

	Active Brains<br>N=3810	Active Brains plus support<br>N=3826
Active Brains use*		
Getting active use, n (%)	1363/2191 (62.2%)	1333/2197 (60.7%)
Strength balance use, n (%)	1237/2187 (56.6%)	1219/2199 (55.4%)
Breaks from sitting use, n (%)	1132/2178 (52.0%)	1152/2196 (52.5%)
Healthy eating, n (%)	1169/2188 (53.4%)	1137/2193 (51.8%)
Problematic Experience of Therapy Scale (PETS)		
Symptoms too severe or aggravated by therapy	339/2207 (15.4%)	328/2210 (14.8%)
Uncertainty about how to carry out the treatment	482/2197 (21.9%)	449/2203 (20.4%)
Doubts about treatment efficacy	883/2198 (40.2%)	865/2200 (39.3%)
Practical problems	1534/2203 (69.6%)	1453/2198 (66.1%)
Social Support for Exercise (SSE) - family, median (IQR)	1.6 (1.2-2.2)	1.6 (1.2-2.3)
Social Support for Exercise (SSE) - friend, median (IQR)	1.3 (1.0-1.8)	1.3 (1.0-1.8)
Perceived Ease of Use (PEU), median (IQR)	67 (50-83)	67 (50-88)

*any self-reported engagement with Active Brains recommendations; PETS - strongly agree or slightly agree at least one question in the domain; SSE ranges from 1 to 5 where higher scores indicate greater social support for physical activity and exercise; PEU ranges from 0 to 100 where higher scores indicate greater perceived ease of use

Table 6. Baseline characteristics by missingness

	Not missing primary outcome N=8243	Missing primary outcome N=3320
Age mean (SD)	70.7 (6.5)	71.3 (6.7)
Sex		
Male	3406 (41.3%)	1460 (44.3%)
Female	4837 (58.7%)	1835 (55.7%)
Ethnicity		
White	7934 (96.3%)	3153 (95.7%)
Mixed	46 (0.6%)	22 (0.7%)
Asian	162 (2.0%)	67 (2.0%)
Black	65 (0.8%)	33 (1.0%)
Other	36 (0.4%)	20 (0.6%)
Living situation		
On my own	2104 (25.5%)	906 (27.5%)
With my spouse/partner	5792 (70.3%)	2228 (67.6%)
With a relative	286 (3.5%)	134 (4.1%)
With a friend	39 (0.5%)	15 (0.5%)
With a housemate	22 (0.3%)	12 (0.4%)
Education		
GCSE/O-Levels	2772 (33.6%)	1208 (36.7%)
A-levels	1414 (17.2%)	592 (18.0%)
Diploma/BTEC	1812 (22.0%)	708 (21.5%)
Undergraduate	1509 (18.3%)	525 (15.9%)
Post-graduate	594 (7.2%)	226 (6.9%)
PhD	142 (1.7%)	36 (1.1%)
Internet use		
Less often	89 (1.1%)	70 (2.1%)
A few times each month	126 (1.5%)	50 (1.5%)
A few times each week	1233 (15.0%)	555 (16.8%)
Every day	6795 (82.4%)	2620 (79.5%)
Brain activities		
Less than once a month	1941 (23.5%)	826 (25.1%)
Once a month	974 (11.8%)	487 (14.8%)
Once every 2 weeks	271 (3.3%)	109 (3.3%)
Once a week	696 (8.4%)	273 (8.3%)
A few times a week	1749 (21.2%)	654 (19.8%)
Every day	2612 (31.7%)	946 (28.7%)
Digit span test baseline, mean (SD)	5.7 (3.0)	5.5 (3.3)
Paired associates baseline, mean (SD)	3.4 (1.1)	3.3 (1.2)
Self ordered search baseline, mean (SD)	5.6 (3.3)	4.9 (3.4)
Verbal reasoning baseline, mean (SD)	15.5 (6.2)	14.4 (6.6)
MCI flag based on verbal reasoning test	5008 (60.8%)	2182 (66.2%)
IQCODE, mean (SD)	3.1 (0.3)	3.1 (0.4)
IADL, mean (SD)	0.9 (2.3)	1.4 (3.2)
SWEMWBS, mean (SD)	25.7 (4.6)	25.4 (5.0)
SF-12 PCS, mean (SD)	42.3 (8.8)	40.6 (9.7)
SF-12 MCS, mean (SD)	50.6 (8.9)	49.6 (9.6)
Geriatric Depression Scale		
Not depressed	4276 (51.9%)	1538 (46.7%)
Uncertain	2271 (27.6%)	873 (26.5%)
Depressed	1696 (20.6%)	884 (26.8%)
IPAQ MET mins, median (IQR)	4026 (2133-6326)	3546 (1639-5839)
Walking minutes, mean (SD)	630 (360-1050)	525 (300-1050)
Self-efficacy for exercise, mean (SD)	54.4 (20.5)	51.1 (22.3)

Locus Causation for Exercise, mean (SD)	4.7 (1.6)	4.5 (1.5)
Current smoker		
No	7944 (96.4%)	3113 (94.5%)
Occasionally	76 (0.9%)	51 (1.5%)
Yes	223 (2.7%)	131 (4.0%)
Currently ever drink alcohol		
No	1996 (24.2%)	903 (27.4%)
Yes	6247 (75.8%)	2392 (72.6%)
Social Support Survey, mean (SD)	77.6 (25.8)	75.2 (26.9)
Prudent diet score, mean (SD)	0.1 (1.2)	0.1 (1.4)
Heart problems	1189 (14.4%)	534 (16.2%)
Heart valve problems	230 (2.8%)	115 (3.5%)
Back/neck pain	2171 (26.3%)	946 (28.7%)
Cancer	635 (7.7%)	299 (9.1%)
Claudication	170 (2.1%)	112 (3.4%)
Crohns/colitis	106 (1.3%)	52 (1.6%)
Depression/anxiety	967 (11.7%)	501 (15.2%)
Diabetes	729 (8.8%)	416 (12.6%)
High blood pressure	3080 (37.4%)	1393 (42.3%)
IBS	626 (7.6%)	274 (8.3%)
Liver problems	159 (1.9%)	75 (2.3%)
Lung problems	908 (11.0%)	436 (13.2%)
Osteoarthritis	2378 (28.8%)	991 (30.1%)
Psychosis	22 (0.3%)	16 (0.5%)
Renal problems	217 (2.6%)	116 (3.5%)
Rheumatoid arthritis	278 (3.4%)	168 (5.1%)
Stroke/TIA	354 (4.3%)	161 (4.9%)
Family history dementia	1879 (25.3%)	697 (23.5%)
Number of medications, median (IQR)	2 (1-5)	3 (1-5)

Digit span test (attention); Paired associates test (verbal short-term memory); Self-ordered search (spatial working memory)
AACD age associated cognitive decline (1SD below norm for verbal reasoning); MCI mild cognitive impairment (1.5SD below norm for verbal reasoning)
IQCODE Informant Questionnaire on Cognitive Decline in the Elderly, 1=much improved to 5 =much worse
IADL Instrumental Activities of Daily Living 0 to 35 with lower scores indicating less help needed.
SWEMWBS Short Warwick Edinburgh Mental Well-Being Scale - Scores range from 7 to 35 and higher scores indicate higher positive mental wellbeing.
EQ-5D health related quality of life
SF-12 Short Form Health Survey Questionnaire – PCS Physical Component Summary, MCS Mental Component Summary range from 0 to 100 with higher scores indicating better health-related quality of life.
GDS Geriatric Depression Scale
IPAQ International Physical Activity Questionnaire – MET minutes metabolic equivalent of time minutes
SEE Self efficacy for exercise ranges from 0 to 90 with higher score indicating greater higher self-efficacy for exercise
LCE Locus of Causation for Exercise - ranges from 1 strongly disagree to 7 strongly agree where high scores indicate greater self-determination
SSS Social Support Survey ranges from 0 to 100 with higher scores indicating greater support
FFQ Food Frequency Questionnaire prudent diet score

Table 7. Protocol changes

Amendment Number	Substantial/Non-Substantial Amendment	Date of Submission to ERGO	Date ERGO approved	Date of submisson to IRAS/HRA	Date HRA approved	Document name	Version date & number	Summary of key changes
1 (.A1)	Substantial	30/03/2020	02/04/2020	07/04/2020	24/04/2020 reissued 2*7/07/20	Protocol	V3 27.03.20	Clarification of follow up procedure; collection of additional clinical measures at baseline and follow-up
8 (.A8)	Non-substantial	07/05/2021	13/05/2021	25/05/2021	25/05/2021	Protocol	V4 29.04.21	Minor wording change: removal of "via Docmail" on page 12
9 (.A9)	Non-substantial	27/05/2021	03/06/2021	04/06/2021	04/06/2021	Protocol	V5 17.05.21	Amendments to processes to manage recruitment of those experiencing cognitive effects of long-covid
11 (.A11)	Non-substantial	08/09/2021	16/09/2021	17/09/2021	17/09/2021	Protocol	V6 18.08.21	Addition of sample size calculation for cognitive impairment (WS1) trial (previously omitted but no change to sample size)
14 (.A14)	Non-substantial	31/05/2022	17/06/2022	18/06/2022	18/06/2022	Protocol	V7 270422	Clarification of sample sizes required for different outcomes and different time points (requested by PSC - no change to sample size)
17 (.A17)	Non-substantial	04/03/2024	18/03/2024	19/03/2024	19/03/2024	Protocol	V8 04.03.24	Clarify secondary research objective and justify change to the alpha value in sample size calculations for Stream 1 (approved by steering committee and NIHR PGfAR) - but with no overall impact on target sample size
18 (.A18)	Substantial	23/09/2024	04/10/2024 & 10/10/24	11/10/2024	24/10/24 (REC/HRA/HCRW)	Protocol	V9 23.09.24	Minor changes to study team/ co-investigator details; updates to list of measures to correct inaccuracies for data collection time points for a couple of measures ; elaboration on process for estimating dementia at longterm follow up; clarification of how 'severe cognitive impairment' will be defined in analysis; proposed additional process for collection of informant IQCODE data to maximise longterm data collection

Sample Size Calculations

Baddeley verbal reasoning test (Primary outcome): To limit the size of the study, with independent comparisons with control it is not mandatory to allow for multiplicity, as is currently the case with a host of platform trials and multi-drug trials which use a common control group [9-11]. We therefore assumed alpha=0.05 for comparisons between each intervention group and control, which was agreed by both the Trial Steering Committee (TSC) and the funder. For the primary outcome of cognitive performance (verbal reasoning) at 12 months, for 80% power to detect a standardised effect of 0.15[12] required 698 per group for the cMCI subgroup and the same again for the cAACD subgroup, or 4188 in total. Assuming 70% could be followed-up at one year, 5983 participants were required in total, and assuming 60% would be followed-up at 5 years, 6980 in total were required.

However, since the limiting factor in the sample size calculation was for the key secondary outcome of dementia at 5 years (see below), the total sample size provided more power for the primary outcome.

Progression to dementia**:** The limiting factor in the sample size calculation was the progression to dementia at five-years. For alpha 0.05 and 80% power, we aim to detect a difference of 5% after 5 years (20% intervention and 25% control) in those with dementia. Therefore, 1094 in each of the cMCI and cAACD groups or a total of 2188 were needed. Assuming that 60% will be available to provide follow up in 5 years, 3647 were needed in each of the 3 study groups or 10940 in total.

Patient and Public involvement.

Public contributors (n = 5; 2 women, 3 men) with full collaborator status helped to develop the protocol, and all materials and processes associated with this study (e.g. participant information leaflets, interview topic guides, follow-up data collection procedures). All public contributors are adults aged 60 years and over, some with experience of caring for close family members with cognitive decline. These public contributors previously inputted on all aspects of the development of the Active Brains intervention, and subsequently the processes and procedures involved in planning and conducting the feasibility trial[19]. As such they have a continuity and depth of understanding of the programme and its objectives that, alongside their personal experience and knowledge, have been highly valuable in guiding their contributions to the conduct of the trial. During the trial, they attended regular progress meetings contributing to management discussions and decisions regarding developing and operationalising the study. They also contributed to the writing up of study outputs including publications and lay summaries. PPI contributors on our trial steering committee (n=2; 1 woman, 1 man) have provided independent guidance throughout the programme, and most recently were involved in reviewing and providing feedback on our statistical analysis plan.

References

1. Baddeley A. A 3 minute reasoning test basedon grammatical transformation. *Psychon Sci* 1968; **10 (10)**: 341–2.
2. Brooker H, Williams G, Hampshire A, et al. FLAME: A computerized neuropsychological composite for trials in early dementia. *Alzheimers Dement (Amst)* 2020; **12**(1): e12098.
3. Furnham A. Measuring Intelligence in 3 Min: Concurrent, Convergent and Discriminant Validity of the Baddeley Reasoning Test*Applied Cognitive Psychology* 2024; **38**: e4244.

4. Ballard CB, H.; Khan,Z.; Corbett,A.; Aarsland,D.;Wesnes,K. P1-658: Repeated cognitive testing rediced sample size necessary to detect clinically relevant effects

https://doi.org/10.1016/j.jalz.2018.06.670C. Alzheimer's Association International Conference: ; 2018 1/7/2018; 2018.
5. Wesselman LM, Hooghiemstra AM, Schoonmade LJ, de Wit MC, van der Flier WM, Sikkes SA. Web-Based Multidomain Lifestyle Programs for Brain Health: Comprehensive Overview and Meta-Analysis. *JMIR mental health* 2019; **6**(4): e12104.
6. Huntley J, Corbett A, Wesnes K, et al. Online assessment of risk factors for dementia and cognitive function in healthy adults. *Int J Geriatr Psychiatry* 2018; **33**(2): e286–e93.
7. Corbett A, Owen A, Hampshire A, et al. The Effect of an Online Cognitive Training Package in Healthy Older Adults: An Online Randomized Controlled Trial. *J Am Med Dir Assoc* 2015; **16**(11): 990–7.
8. Wesnes KA, Brooker H, Ballard C, McCambridge L, Stenton R, Corbett A. Utility, reliability, sensitivity and validity of an online test system designed to monitor changes in cognitive function in clinical trials. *Int J Geriatr Psychiatry* 2017; **32**(12): e83–e92.
9. Stallard N, Todd S, Parashar D, Kimani PK, Renfro LA. On the need to adjust for multiplicity in confirmatory clinical trials with master protocols. *Ann Oncol* 2019; **30**(4): 506–9.
10. Woodcock J, LaVange LM. Master Protocols to Study Multiple Therapies, Multiple Diseases, or Both. *N Engl J Med* 2017; **377**(1): 62–70.
11. Cook R, Farewell V. Multiplicity Considerations in the Design and Analysis of Clinical Trials. *J Royal Stats Soc* 1996; **Series A 159**: 93–110.
12. Zhang H, Huntley J, Bhome R, et al. Effect of computerised cognitive training on cognitive outcomes in mild cognitive impairment: a systematic review and meta-analysis. *BMJ Open* 2019; **9**(8): e027062.
13. Mainzer RM, Nguyen CD, Carlin JB, Moreno-Betancur M, White IR, Lee KJ. A comparison of strategies for selecting auxiliary variables for multiple imputation. *Biom J* 2024; **66**(1): e2200291.
14. Sullivan TR, White IR, Salter AB, Ryan P, Lee KJ. Should multiple imputation be the method of choice for handling missing data in randomized trials? *Stat Methods Med Res* 2018; **27**(9): 2610–26.
